# Attitudes and Beliefs Related to Low Back Pain and Associated Clinical and Psychosocial Factors Among Adults Receiving Physiotherapy in Jordan: A Cross-Sectional Study

**DOI:** 10.2147/JPR.S633453

**Published:** 2026-10-22

**Authors:** Saddam F Kanaan, Hadeel Alissa, Ala’a Fayez Jaber, Fuad Asem Kanaan, Khader Almhdawi, Jennifer Kirsten Allen

**Affiliations:** 1 Department of Rehabilitation Sciences, College of Health Sciences, QU Health, Qatar University, Doha, Qatar; 2 Department of Rehabilitation Sciences, Jordan University of Science and Technology, Irbid, Jordan; 3 Faculty of Medicine, Jordan University of Science and Technology, Irbid, Jordan

**Keywords:** low back pain, back-pain attitudes questionnaire, fear avoidance, biopsychosocial model, disability, psychological distress, Jordan

## Abstract

**Aim:**

To describe attitudes and beliefs about low back pain (LBP) among adults receiving physiotherapy care in Jordan and to identify demographic, clinical, disability, psychological distress, and fear-avoidance factors associated with Back-Pain Attitudes Questionnaire (Back-PAQ) total scores.

**Materials and Methods:**

This cross-sectional study included adults with acute or chronic LBP. Sociodemographic and clinical data were collected using interviewer-administered, face-to-face questionnaires. Standardized measures included the Back-PAQ to assess attitudes toward LBP; the Fear-Avoidance Beliefs Questionnaire (FABQ); the Oswestry Disability Index (ODI); the Depression, Anxiety, and Stress Scales (DASS); and the Numeric Pain Rating Scale (NPRS).

**Results:**

A total of 269 participants were included (mean age 39.86±11.27 years); the mean Back-PAQ total score was 131.66±11.82. Only 14.9% endorsed staying active during an episode, whereas 82.9% believed that the back is easily injured and 92.6% believed that it could be injured without care. Higher Back-PAQ scores were associated with female gender (B= 5.166, p=0.001), longer LBP duration (B=0.039, p=0.006), moderate versus mild pain intensity (B=3.920, p=0.021), and higher FABQ-Physical Activity scores (B=0.387, p<0.001).

**Conclusion:**

Patients with LBP endorsed unhelpful beliefs about back vulnerability, pain causing harm, and restricting activity due to pain. Attitudes toward LBP were largely negative. These findings describe associations in a treatment-seeking sample. The cross-sectional design does not support causation, and longitudinal studies are needed to establish temporal relationships.

## Introduction

Low back pain (LBP) is a global cause of disability, contributing to activity limitations, work absences, and economic burdens.^1^,^2^ Individuals with LBP may have recurring episodes, with 24% to 80% experiencing recurrences within a year^3^ and 5–10% may develop persistent symptoms of LBP with substantial effects on function and quality of life.^3–5^ The burden of LBP in the Middle East and North Africa region is substantial and is the largest musculoskeletal cause of disability adjusted life years, particularly in Jordan, which is among the highest countries in the region for age-standardized musculoskeletal disability.^6^

LBP management has increasingly shifted beyond purely biomedical explanations to more comprehensive models. Accordingly, contemporary rehabilitation approaches increasingly emphasize the biopsychosocial model, in which pain experience, disability, recovery expectations, emotional distress, and activity behavior are considered together.^7^ Current LBP guidelines recommend education, self-management, and continued activity and exercise, while addressing psychosocial factors.^8^,^9^ In this context, reassurance is an element of evidence-based pain education: it communicates the low likelihood of serious pathology after appropriate assessment; a generally favorable prognosis; the safety of gradual activity; and the distinction between pain and actual tissue damage.^8^,^9^

Within this framework, patients’ beliefs and attitudes are clinically relevant because fear-avoidance, low recovery expectations, and related psychological factors are associated with persistent pain, disability, and activity and participation restriction.^8–10^ The fear-avoidance model suggests that catastrophic beliefs about pain can contribute to pain-related fear and avoidance; less catastrophic beliefs about pain can contribute to feeling comfortable confronting pain and engaging in activity.^11^ Key attitudes towards LBP involve beliefs regarding back vulnerability, association of pain with injury, necessity for protection, prognosis, and activity during pain episodes.^12–14^

Such beliefs can shape perceptions of illness severity, physical and psychological functioning, coping mechanisms, recovery expectations and treatment adherence.^10^,^15^,^16^ Unhelpful or maladaptive beliefs have been associated with greater disability, fear-avoidant behaviors, and poorer recovery, supporting further study of these beliefs in clinical populations.^10^ Educational and cognitive-behavioral strategies may address misconceptions about pain, fear of harm, and confidence in movement. Therefore, understanding factors associated with patients’ beliefs is fundamental to informing hypotheses for future intervention studies.^17^

Several instruments have been developed to assess attitudes and beliefs about LBP across clinical and community populations. The Back-Pain Attitudes Questionnaire (Back-PAQ) was developed for the public, people with back pain, and healthcare professionals, and evaluates beliefs concerning back vulnerability, protection, pain-injury links, activity during pain, prognosis, and psychological influences on recovery. It has been translated and adapted for Arabic-speaking populations.^18^ Studies on attitudes toward LBP among patients with LBP are scarce, with few employing a standardized, pre-established, valid and reliable assessment tool such as Back-PAQ.^12^ Patient-based Back-PAQ studies in Arabic-speaking Middle Eastern populations, in general and in clinical settings, are scarce. Also, there are limited studies investigating the association of the Back-PAQ with clinically relevant variables such as symptom duration, pain-intensity categories, disability, psychological distress and fear-avoidance beliefs.

Therefore, this study aimed to describe attitudes and beliefs toward LBP among adults receiving physiotherapy care in Jordan, using the Back-PAQ. We also examined factors independently associated with Back-PAQ total scores, including demographic, clinical, pain-related, disability, psychological, and fear-avoidance factors. This study presents patient-based evidence from an Arabic-speaking Middle Eastern physiotherapy setting, where evidence on patient beliefs and their correlates remains limited. Because of the descriptive, associative design, the findings do not constitute results for screening performance, prognosis, treatment response or intervention effectiveness.

## Methods

### Study Design and Participants’ Recruitment

We conducted a clinic-based cross-sectional study among adults attending physiotherapy departments in university, Ministry of Health, and military hospitals, and private clinics in the North, Middle, and South regions of Jordan, with acute or chronic LBP. Recruitment used a non-probability convenience sampling approach with regional and sector targets intended to broaden coverage. The data were collected from November 2019 to July 2021. The sample was not probability-based or nationally representative. No screening log was maintained; therefore, we could not determine the numbers approached, excluded before enrolment, or declining participation, or the response rate. All 269 participants were also included in the prior knowledge publication; the present analysis addresses Back-PAQ attitudes rather than LBP knowledge.^19^ Region denotes participants’ residence, derived from governorate, rather than recruitment-site location.

### Inclusion and Exclusion Criteria

Adults aged 18–65 years with acute or chronic LBP, as diagnosed by a specialist physician (orthopedist or neurologist), were eligible. Patients were excluded if they were healthcare professionals; were unable to read Arabic; were pregnant; had scoliosis, abdominal aortic aneurysm, malignancy, fracture, spinal infection, epidural abscess, or cauda equina syndrome; had a diagnosed psychiatric disorder requiring ongoing treatment; or had an apparent cognitive or communication impairment. Health professionals were excluded because professional training could influence LBP beliefs.

Jordan has an estimated five million adults aged 18–65 years.^20^ Applying the global point prevalence of LBP of 7.5%, an estimated 375,000 adults in the country have LBP.^21^ Using an expected prevalence of 7.5%, a 95% confidence level, and 5% absolute precision, the minimum sample required for the descriptive objective was 107 participants. Because we used multiple linear regression, an a priori G*Power calculation indicated that a minimum of 172 participants were required for a medium effect (f^2^=.15), 10 possible associated factors, α=0.05, and statistical power of 95%.^22^ The final sample of 269 exceeded both targets. Participants were selected based on the population’s distribution across three main regions: Northern (28.6%), Middle (63.5%), and Southern (7.9%). Regional population proportions served as coverage targets, not probability-sampling fractions.

### Data Collection Procedure

Four trained research assistants (including three physiotherapists and one paramedic) conducted face-to-face interviews and recorded responses on structured paper questionnaires. Training addressed effective interview techniques and wording, neutral prompting, questionnaire instructions, and completeness checks. The data collected included demographic, socioeconomic, behavioral, and clinical characteristics, as well as responses to standardized questionnaires. All questionnaires were administered in Arabic using validated Arabic versions where available. The interviewer-administered format was intended to reduce response omissions, support understanding of questionnaire instructions, and standardize data collection procedures across sites. Data were checked for completeness and plausibility before analysis.

### Outcome Measures

A structured questionnaire with 18 items assessed sociodemographic, behavioral and clinical characteristics: age, body mass index, LBP duration, number of physical therapy sessions, gender, marital status, number of children, region, healthcare sector, insurance, household location, employment status, education level, work demands, monthly income, smoking, comorbidities, painkiller use, and weekly exercise. We selected these variables to characterize the treatment-seeking sample and examine plausible social, occupational, behavioral, and clinical correlates of LBP beliefs. The Arabic version of the 34-item Back-Pain Attitudes Questionnaire (Back-PAQ) assessed beliefs about back vulnerability, protection, pain, activity, prognosis, and psychological influences.^12^,^18^ Items are rated on a five-point scale from “False” to “True.” Items 1, 2, 3, 15, 16, 17, 27, 28, 29, 30, and 31 were reverse-scored; higher total scores (range 34–170) represent more unhelpful beliefs. The 34-item Back-PAQ had acceptable internal consistency (α = 0.70) in its original general-population development study.^12^ In this sample, Cronbach’s α was 0.713. In the present study, the Back-PAQ total score was treated as the main dependent variable.

The Arabic version of the Depression, Anxiety and Stress Scale (DASS-21) assessed the psychological symptoms of depression, anxiety, and stress.^23^ DASS-21 is a valid and reliable self-assessment mental health tool with three self-reported subscales (seven items each) for depression, anxiety, and stress. Scores range from 0 to 21, with higher scores indicating more severe mental health symptoms. Raw subscale scores were not doubled in these analyses. The internal consistency for DASS-21 subscales is excellent, with Cronbach’s α = 0.94, 0.87 and 0.91 for depression, anxiety and stress, respectively.^24^ Current-sample α values are 0.803 for depression, 0.825 for anxiety and .860 for stress (n=269).

The Arabic version of the Fear-Avoidance Beliefs Questionnaire (FABQ) was used to assess fear-avoidance beliefs about physical activity and work on LBP and the resulting disability. The FABQ is a valid and reliable questionnaire (ICC = 0.97), which consists of 16 items, divided into two subscales: a work scale (items 6, 7, 9, 10, 11, 12, 15) with a maximum score of 42, and a physical activity scale (items 2, 3, 4, 5), with a maximum score of 24.^25^ Higher FABQ subscale scores indicate stronger fear-avoidant beliefs. FABQ-Physical Activity and FABQ-Work were scored and analyzed separately, because they represent related but distinct fear-avoidance domains.

The Arabic version of the Oswestry Disability Index (ODI) was used to assess functional disability. ODI is a valid, reliable assessment tool with acceptable internal consistency (Cronbach’s α ranging from 0.71 to 0.87) and test-retest reliability (r =0.83-0.99); it varies with the interval between measurements. It collects information about the effect of LBP on an individual’s ability to manage everyday life. Intraclass correlation coefficient values for the ODI range from 0.84 to 0.94. It includes 10 sections, each with a total possible score of five.^26^ Current study Cronbach’s α= 0.721.

The Numeric Pain Rating Scale (NPRS) was used to assess an individual’s level of pain intensity. The NPRS is a valid, reliable, quick and easy-to-use tool for assessing pain intensity. It consists of a numeric rating scale with response options ranging from “no pain,” which corresponds to a “0 score,” to “worst pain ever,” illustrated by a “10 score”.^27^ For categorical analyses, NPRS scores were further classified as mild pain (<4), moderate pain (4.0–6.9), and severe pain (≥7.0).^28^

### Ethical Considerations

The Institutional Review Board (IRB) at Jordan University of Science and Technology (IRB approval #: 669–2019) and the Ministry of Health approved this study. Participants were given detailed information about the study. Informed oral and written consent was obtained from each patient before participating in the study. Patients were informed that their participation was voluntary and that they were free to withdraw from the study at any time without explanation or negative consequences related to their standard treatment. Confidentiality was maintained through participant identification numbers and secure data storage. The study was conducted in accordance with the Declaration of Helsinki.

### Statistical Analysis

Descriptive statistics were presented as means and standard deviations. Categorical variables were converted into dummy variables. Item-level Back-PAQ responses were summarized using means with 95% confidence intervals and the percentage of participants endorsing “possibly true” or “true.” The univariate normality distribution of continuous variables was also assessed. The multivariable model used 240 complete cases; 29 participants had one or more missing values. Missing values were 19 age, 20 BMI, and 10 LBP duration values. Assumptions were evaluated using residual-versus-fitted and Q–Q plots, a studentized Breusch–Pagan test, and variance inflation factors (VIF >5 indicating multicollinearity). Influential observations were examined using leverage and Cook’s distance ((a sensitivity analysis excluded observations with Cook’s distance >4/n)). We employed forward and backward selection in multivariable linear regression models to identify the optimal model that best fit the data, with the Back-PAQ total score as the dependent variable. The analysis was conducted using SPSS version 31.0, with a significance level set at p<0.05.

## Results

### Participant Characteristics

A total of 269 individuals with LBP, with an average age of 39.86 years (±11.27), completed the study. The mean body mass index was 26.42 ± 4.09 kg/m^2^, and the mean duration of LBP was 37.61 ± 55.24 months. More than half of the participants were women (56.9%), 66.9% were married, 55.0% were employed, and 77.7% had health insurance. Table 1 presents the participants’ demographic, socioeconomic, behavioral, and clinical characteristics.

**Table 1:** Sociodemographic and Clinical Characteristics of the Participants (N = 269)

Characteristics	Mean (SD) or n (%)
Age (years)	39.86 (11.27)
Body mass index (kg/m^2^)	26.42 (4.09)
Low back pain duration (months)	37.61 (55.24)
Total number of physical therapy sessions	7.50 (10.10)
**Gender**	
Male	116 (43.1%)
Female	153 (56.9%)
**Marital status**	
Single/divorced/widowed	87 (32.3%)
Married	180 (66.9%)
Missing	2 (0.7%)
**Number of children**	
0	78 (29.0%)
1–5	104 (38.7%)
≥6	32 (11.9%)
Missing	55 (20.4%)
**Region (derived from governorate)**	
North	141 (52.4%)
Middle	100 (37.2%)
South	28 (10.4%)
**Health care sector**	
Ministry of Health	131 (48.7%)
Royal Medical Services	42 (15.6%)
University hospital	51 (19.0%)
Private sector	41 (15.2%)
Missing	4 (1.5%)
**Health insurance**	
Insured	209 (77.7%)
Uninsured	48 (17.8%)
Missing	12 (4.5%)
**Household location**	
Urban	87 (32.3%)
Suburban	114 (42.4%)
Rural areas	68 (25.3%)
**Highest education level**	
High school	161 (59.9%)
Diploma	41 (15.2%)
Bachelor’s or higher	65 (24.2%)
Missing	2 (0.7%)
**Employment**	
Employed	148 (55.0%)
Unemployed	121 (45.0%)
**Work demands**	
Physical demands	72 (26.8%)
Mental demands	21 (7.8%)
Both	152 (56.5%)
Missing	24 (8.9%)
**Monthly income (Jordanian Dinar)**	
<250	48 (17.8%)
250–460	95 (35.3%)
>460	112 (41.6%)
Missing	14 (5.2%)
**Smoking status**	
Smoker	90 (33.5%)
Non-smoker	178 (66.2%)
Missing	1 (0.4%)
**Comorbidities**	
Yes	56 (20.8%)
No	212 (78.8%)
Missing	1 (0.4%)
**Use of painkillers**	
Yes	108 (40.1%)
No	161 (59.9%)
**Exercise hours per week**	
No exercise	163 (60.6%)
One hour a week	67 (24.9%)
More than an hour a week	39 (14.5%)

**Notes**: Continuous variables are reported as mean (SD). Categorical variables are presented as n (%).

**Abbreviations**: SD, standard deviation; BMI, body mass index; LBP, low back pain.

### Back-PAQ Item and Scale Responses

Item-level Back-PAQ responses are presented in Table 2. For ease of interpretation, “Possibly true” and “True” responses were combined. For most items, endorsement of “Possibly true/True” reflects more unhelpful beliefs about LBP. For reverse-worded or positively framed items, endorsement reflects more adaptive beliefs. Items 1, 2, 3, 15, 16, 17, 27, 28, 29, 30, and 31 were reverse-scored when calculating the total Back-PAQ score. The clearest descriptive pattern concerned vulnerability and pain-related harm. Few participants (5.9%) endorsed their back as one of the strongest parts of their body (item 1), or well designed for daily use (7.8%), whereas beliefs that painful movement should be avoided (88.5%) and that ignoring pain could cause injury (85.5%) were also common. Full item estimates and confidence intervals are provided in Table 2.

**Table 2:** Likert Means of the Back-PAQ with 95% CI, and True/Possibly True Endorsement Percentage

Item	Statement	Mean (95% CI)	n (%) Endorsing True/Possibly True
LBA1*	Your back is one of the strongest parts of your body	1.52 (1.40–1.64)	16 (5.9%)
LBA2*	Your back is well designed for the way you use it in daily life	1.61 (1.48–1.75)	21 (7.8%)
LBA3*	Bending your back is good for it	2.57 (2.37–2.76)	79 (29.4%)
LBA4	Sitting is bad for your back	3.76 (3.60–3.92)	175 (65.1%)
LBA5	Lifting without bending the knees is not safe for your back	4.23 (4.09–4.38)	220 (81.8%)
LBA6	It is easy to injure your back	4.27 (4.14–4.39)	223 (82.9%)
LBA7	It is important to have strong muscles to support your back	4.47 (4.38–4.57)	239 (88.8%)
LBA8	Good posture is important to protect your back	4.65 (4.58–4.73)	256 (95.2%)
LBA9	If you overuse your back, it will wear out	4.51 (4.42–4.60)	247 (91.8%)
LBA10	If an activity or movement causes back pain, you should avoid it in the future	4.48 (4.38–4.58)	238 (88.5%)
LBA11	You could injure your back if you are not careful	4.60 (4.52–4.68)	249 (92.6%)
LBA12	You can injure your back and only become aware of the injury sometime later	4.32 (4.21–4.42)	224 (83.3%)
LBA13	Back pain means that you have injured your back	4.19 (4.06–4.32)	215 (79.9%)
LBA14	A twinge in your back can be the first sign of a serious injury	3.72 (3.59–3.86)	170 (63.2%)
LBA15*	Thoughts and feelings can influence the intensity of back pain	2.43 (2.28–2.59)	43 (16.0%)
LBA16*	Stress in your life (financial, work, relationship) can make back pain worse	2.34 (2.19–2.49)	41 (15.2%)
LBA17*	When you have back pain, you can do things which increase your pain without harming the back	2.72 (2.56–2.89)	68 (25.3%)
LBA18	Having back pain makes it difficult to enjoy life	4.42 (4.32–4.53)	235 (87.4%)
LBA19	It is worse to have pain in your back than your arms or legs	3.91 (3.77–4.06)	183 (68.0%)
LBA20	It is hard to understand what back pain is like if you have never had it yourself	4.39 (4.27–4.51)	224 (83.3%)
LBA21	If your back hurts, you should take it easy until the pain goes away	3.55 (3.38–3.72)	171 (63.6%)
LBA22	If you ignore back pain, you may cause damage to your back	4.43 (4.33–4.54)	230 (85.5%)
LBA23	It is important to see a health professional when you have back pain	4.52 (4.43–4.61)	243 (90.3%)
LBA24	To effectively treat back pain, you need to know exactly what is wrong	4.52 (4.44–4.61)	238 (88.5%)
LBA25	If you have back pain, you should avoid exercise	3.07 (2.90–3.24)	112 (41.6%)
LBA26	When you have back pain the risks of vigorous exercise outweigh the benefits	4.01 (3.88–4.13)	185 (68.8%)
LBA27*	If you have back pain, you should try to stay active	2.29 (2.14–2.44)	40 (14.9%)
LBA28*	Most back pain settles quickly, at least enough to get on with normal activities	2.33 (2.19–2.47)	32 (11.9%)
LBA29*	Worrying about your back can delay recovery from back pain	2.24 (2.11–2.37)	23 (8.6%)
LBA30*	Focusing on things other than your back helps you to recover from back pain	2.39 (2.25–2.54)	36 (13.4%)
LBA31*	Expecting your back pain to get better helps you to recover from back pain	1.93 (1.81–2.04)	15 (5.6%)
LBA32	Once you have had back pain, there is always a weakness	4.03 (3.90–4.15)	205 (76.2%)
LBA33	There is a high chance that an episode of back pain will not resolve	3.51 (3.36–3.67)	156 (58.0%)
LBA34	Once you have a back problem, there is not a lot you can do about it	3.46 (3.29–3.62)	141 (52.4%)

**Notes**: Responses range from False (1) to True (5). *Positively framed/reverse-scored item; endorsement of True/Possibly true is adaptive for these items. All other item endorsements are interpreted as more unhelpful beliefs.

**Abbreviation**: CI, confidence interval.

Similarly, activity and care-seeking endorsement were restrictive. Only 14.9% supported remaining active during a back pain episode, whereas 63.6% endorsed taking it easy, 68.8% believed the risks of vigorous exercise exceeded the benefits, and 41.6% thought exercise should be avoided. Most participants endorsed seeing a health professional (90.3%) and knowing exactly what was wrong (88.5%) as necessary for effective treatment.

The mean Back-PAQ total score was 131.66±11.82 on the possible 34–170 scale. Because no validated clinical cutoff was identified, the total was not classified as “high” or “low”; interpretation emphasizes the observed score and item-response pattern. Table 3 presents descriptive statistics for pain intensity, DASS-21, FABQ, and ODI.

**Table 3:** Descriptive Statistics of Standardized Outcome Measures’ Scores (n = 269)

Characteristic		Mean (SD) or n (%)
Back-Pain Attitudes Questionnaire total score		131.66 (11.82)
Numeric Pain Rating Scale	Mild pain (0–3.9)	82 (30.5%)
	Moderate pain (4.0–6.9)	128 (47.6%)
	Severe pain (≥7.0)	59 (21.9%)
DASS_Stress		8.55 (5.58)
DASS_Anxiety		6.63 (5.20)
DASS_Depression		6.76 (4.86)
FABQ_Work		21.58 (11.98)
FABQ_Physical activity		14.80 (7.60)
Oswestry Disability Index (ODI)		30.75 (14.54)

**Notes**: Continuous variables are reported as mean (SD); pain-intensity categories are reported as n (%).

**Abbreviations**: SD, standard deviation; Back-PAQ, Back-Pain Attitudes Questionnaire; NPRS, Numeric Pain Rating Scale; FABQ, Fear-Avoidance Beliefs Questionnaire; ODI, Oswestry Disability Index; DASS, Depression Anxiety Stress Scales.

### Factors Associated with the Back-PAQ Total Score

Univariable linear regression was based on 245–269 available cases and is presented in Table 4. Variables were included in the multivariable model on clinical grounds rather than a univariable p-value threshold. Table S1 presents the Pearson correlation matrix.

**Table 4:** Univariable Linear Regression of Back-Pain Attitudes Questionnaire Total Score

Covariate/Contrast	n	B	95% CI	p
**Age, per year**	250	0.155	0.016 to 0.293	0.029
**BMI, per kg/m^2^**	249	0.440	0.079 to 0.801	0.017
**LBP duration, per month**	259	0.055	0.032 to 0.077	<0.001
**Physiotherapy sessions**	260	0.037	−0.151 to 0.224	0.701
**Female vs male**	269	4.316	1.430 to 7.202	0.004
**Single/divorced/widowed vs married**	267	−1.105	−4.312 to 2.103	0.498
**Middle vs North region**	269	−4.062	−6.955 to −1.168	0.006
**South vs North region**	269	−4.756	−11.518 to 2.006	0.167
**Suburban vs urban**	269	−2.537	−5.881 to 0.807	0.136
**Rural vs urban**	269	0.687	−3.013 to 4.386	0.715
**Diploma vs high school**	267	0.859	−3.095 to 4.813	0.669
**Bachelor or higher vs high school**	267	−0.194	−3.296 to 2.907	0.902
**Employed vs unemployed**	269	−3.048	−5.892 to −0.205	0.036
**Physical vs combined work demands**	245	−3.931	−7.575 to −0.288	0.035
**Mental vs combined work demands**	245	−2.475	−7.946 to 2.996	0.374
**Income 250–460 vs <250 JOD**	255	0.094	−4.354 to 4.541	0.967
**Income >460 vs <250 JOD**	255	−0.592	−4.901 to 3.717	0.787
**Insured vs uninsured**	257	−1.980	−5.714 to 1.753	0.297
**Smoker vs non-smoker**	268	−1.714	−4.848 to 1.421	0.283
**Comorbidity yes vs no**	268	2.032	−1.412 to 5.476	0.246
**Painkiller use yes vs no**	269	3.737	0.863 to 6.610	0.011
**Exercise 1 h/week vs none**	269	−1.947	−4.947 to 1.054	0.203
**Exercise >1 h/week vs none**	269	−1.401	−6.331 to 3.529	0.576
**Moderate vs mild pain**	269	4.620	1.388 to 7.852	0.005
**Severe vs mild pain**	269	4.226	0.213 to 8.240	0.039
**DASS stress, per raw point**	269	0.431	0.189 to 0.673	<0.001
**DASS anxiety, per raw point**	269	0.436	0.193 to 0.679	<0.001
**DASS depression, per raw point**	269	0.372	0.096 to 0.648	0.008
**FABQ-Work, per point**	269	0.177	0.065 to 0.290	0.002
**FABQ-Physical Activity, per point**	269	0.527	0.367 to 0.688	<0.001
**ODI, per percentage point**	269	0.083	−0.007 to 0.173	0.070

**Abbreviations**: B, unstandardized coefficient; CI, confidence interval; BMI, Body Mass Index; Back-PAQ, Back-Pain Attitudes Questionnaire; LBP, low back pain; JOD, Jordanian Dinar; FABQ, Fear-Avoidance Beliefs Questionnaire; ODI, Oswestry Disability Index; DASS-21, Depression Anxiety Stress Scale-21.

Regression diagnostics indicated no major assumption violations (VIFs 1.21–3.65). Sensitivity analyses showed that the moderate-pain association weakened after excluding influential observations (p =0.055), and the duration association was not significant after log transformation (p =0.293). In the final multivariable linear regression model, four variables were independently associated with the Back-PAQ total score. The model explained 25.1% of the variance in the Back-PAQ total score and was statistically significant (n = 240; R^2^ =0.251, adjusted R^2^ =0.212; robust F (12, 227) = 7.355, p <0.001). Higher Back-PAQ scores were associated with female gender (B = 5.166, 95% CI: 2.025 to 8.307, p =0.001), longer LBP duration (B = 0.039, 95% CI: 0.012 to 0.067, p =0.006), moderate pain intensity compared with mild pain intensity (B = 3.920, 95% CI: 0.587 to 7.253, p =0.021), and higher FABQ-Physical Activity scores (B = 0.387, 95% CI: 0.193 to 0.580, p <0.001). ODI, DASS-21 subscale scores, and FABQ-Work were not significantly associated with Back-PAQ scores in the final model (Table 5).

**Table 5:** Multivariable Linear Regression of Factors Independently Associated with Back-Pain Attitudes Questionnaire Total Score (n=240)

Covariate	B	95% CI	p
**Intercept**	110.345	101.064 to 119.625	<0.001
**Age, per year**	−0.003	−0.152 to 0.146	0.966
**Female vs male**	5.166	2.025 to 8.307	0.001
**BMI, per kg/m^2^**	0.233	−0.120 to 0.587	0.195
**LBP duration, per month**	0.039	0.012 to 0.067	0.006
**Moderate vs mild pain**	3.920	0.587 to 7.253	0.021
**Severe vs mild pain**	−2.634	−7.723 to 2.455	0.309
**DASS stress, per raw point**	0.228	−0.224 to 0.680	0.322
**DASS anxiety, per raw point**	0.110	−0.422 to 0.641	0.685
**DASS depression, per raw point**	−0.216	−0.745 to 0.314	0.423
**FABQ-Work, per point**	0.071	−0.059 to 0.201	0.282
**FABQ-Physical Activity, per point**	0.387	0.193 to 0.580	<0.001
**ODI, per percentage point**	0.046	−0.080 to 0.171	0.474

**Notes**: Moderate pain intensity was coded as NPRS =4.0–6.9. Complete-case model n=240; R^2^=.251; adjusted R^2^=.212; robust F(12,227)=7.355; p<0.001.

**Abbreviations**: BMI, Body Mass Index; LBP, Low Back Pain; DASS-21, Depression Anxiety Stress Scale-21; ODI, Oswestry Disability Index; FABQ, Fear-Avoidance Beliefs Questionnaire; CI, confidence interval; *B*, unstandardized coefficient.

## Discussion

This cross-sectional study found that most adults receiving physiotherapy care for LBP in Jordan endorsed beliefs about back vulnerability and activity restriction. In the multivariable model, more negative attitudes were associated with female gender, longer duration of LBP, moderate pain intensity, and higher fear-avoidance beliefs related to physical activity. These findings suggest that Back-PAQ scores are associated with selected clinical characteristics and activity-related beliefs; directionality cannot be determined.

The Back-PAQ results indicated that most participants held negative perceptions of LBP, viewing the back as vulnerable, easily injured and requiring stronger muscles and protection. This interpretation is reinforced by high endorsement levels for items related to back fragility, pain as a sign of harm, avoidance of painful movements and the need for medical consultation. Based on recent Back-PAQ network analysis, belief domains that are most central in people with and without LBP include beliefs about back damage, exercise, and exercise avoidance, which should be important targets for patient education and counseling.^29^

The percentage of respondents affirming the statement “the back is one of the strongest parts of the body” (5.9%) was markedly low compared to the proportions of participants holding this belief in the United Kingdom (60%), New Zealand (76%), and Argentina (62.7%).^30–32^ Notably, these studies used public, community, or general population samples, whereas our study included participants with LBP visiting physiotherapy clinics. Differences may therefore reflect sampling, care-seeking status, health-system messaging, exposure to pain education, translation or administration, and sociocultural beliefs. Direct cross-country comparisons should therefore be interpreted cautiously. This suggests that most participants believed the spine is fragile rather than strong and adaptable, which may be associated with protective behavior and reduced confidence in movement; these mechanisms were not tested. Therefore, education about LBP should emphasize that the spine is a strong and adaptable structure, and an LBP episode does not necessarily mean there is serious tissue damage.^33^

The majority of the sample believed that back protection is important, as indicated by items 5, 6, 7, 8, and 9. Consistent with the results reported by Darlow et al and Pierobon et al, these findings may reflect long-held social conventions.^31^,^32^ Although many participants believed that posture and lifting techniques are important for protecting their backs, there is no conclusive evidence that these factors are a significant cause of LBP. Moreover, the belief that the spine should be in optimal posture and that correct lifting techniques should be used could reinforce fear-avoidance and constant thoughts about the need to protect the back.^32^,^34^ These messages should be reframed toward confidence, graded movement, and individualized activity modification rather than rigid avoidance.

Participants showed limited recognition of the role of psychological and emotional factors in LBP. Few participants endorsed the role of thoughts, feelings, stress or worry in pain and recovery. These proportions are lower than those reported in Belgium, New Zealand, and Argentina.^31^,^32^,^35^ This is relevant because current guidelines suggest a biopsychosocial approach to LBP, which takes into account the physical, psychological and social components of the problem rather than a purely structural approach. However, in the present study, DASS stress, anxiety, and depression were not independently associated with Back-PAQ scores in the final multivariable model. These findings do not establish that psychological factors are unimportant; unmeasured constructs, shared variance and measurement error may influence these estimates. Compared to the populations of New Zealand (80%), Canada (56%), and Argentina (62.4%), only 14.9% of our respondents believe they should remain active while experiencing back pain.^31^,^32^,^36^ Meanwhile, 41.6% believed exercise should be avoided, and 68.8% believed the risks of vigorous exercise outweighed the benefits. This pattern is directly consistent with the final regression model, where higher FABQ-Physical Activity scores were independently associated with more negative Back-PAQ scores. Current guidelines consistently recommend education, staying active, exercise, avoidance of bed rest, and self-management for LBP.^9^ Therefore, activity-related fear-avoidance should be a central focus of patient education in this population, especially because FABQ-Physical Activity was independently associated with Back-PAQ scores in the final model.^17^

Participants frequently endorsed the need for a health professional and an exact diagnosis. This is in line with the results in New Zealand and Argentina.^31^,^32^ This suggests that a biomedical model orientation remains strong in healthcare service delivery and patient orientation. Clinical assessment remains important for identifying serious pathology; separately, most LBP is classified as nonspecific, and effective management does not require identification of a single anatomical pain generator.^8^,^9^,^37^ Overemphasis on identifying an exact anatomical source may reinforce worry and delay active self-management.

Only 11.9% believed back pain resolves quickly, contrasting with 44% in the UK, 45% in Argentina, and 64% in New Zealand, indicating a negative view on prognosis.^30–32^ Moreover, 76.2% worried about lasting weakness from back pain, compared to 30.2% in Argentina and 29% in New Zealand.^31^,^32^ Healthcare providers should address these pessimistic recovery beliefs directly by providing reassurance about the generally favorable course of most LBP episodes, explaining that pain does not always indicate damage, and encouraging gradual return to usual activities. This is especially important because recent longitudinal evidence suggests that negative back beliefs may increase the likelihood of future high-intensity LBP and disability.^38^

The majority of participants were women (56.9%), consistent with evidence that LBP prevalence is often higher among women than men. Gender differences may relate to biological factors (eg, pregnancy-related mechanical and hormonal changes), occupational and caregiving demands, and psychosocial determinants that influence pain experience and care-seeking.^39^ In our study, female gender was independently associated with more negative attitudes toward LBP. This finding may reflect greater perceived vulnerability, higher pain-related concerns, and more persistent disability reported among women with LBP in previous studies.^40^ Recent reviews emphasize that gender can influence the presentation and management of LBP, supporting the need for individualized education and reassurance.^39^ The observed gender association does not identify a biological or social mechanism, and the study did not measure the proposed explanations.

Participants with moderate pain intensity had higher Back-PAQ scores than those with mild pain. Participants with moderate pain may have been more likely to interpret pain as an indicator of injury, and the same can be true for their beliefs about their back as being vulnerable, and their behaviors as being protective, which will serve to reinforce these beliefs. These beliefs align with the Fear-Avoidance Model, which suggests that catastrophizing thoughts about pain may lead to pain-related fear, avoidance, and disability.^10^,^14^ Because this study was cross-sectional, the direction of this association cannot be determined. Negative beliefs may contribute to heightened pain experiences, while greater pain may also reinforce maladaptive beliefs about the back and recovery.^41^ Moreover, two longitudinal studies have demonstrated a significant positive association between negative beliefs about LBP and the continuation of pain.^12^ These findings can be interpreted through a key element of the Fear Avoidance Model of Chronic Pain, which suggests that psychological factors can amplify pain perceptions, causing fear and avoidance, thus reducing functionality.^41^ Whether addressing these thoughts changes outcomes in this sample requires intervention research.^41^

Higher FABQ-Physical Activity scores were independently associated with more negative Back-PAQ scores, suggesting that beliefs about movement and activity are central to patients’ negative attitudes toward LBP. A survey by Darlow et al in New Zealand echoed this, finding that many individuals believe avoiding movement can prevent back injuries, thereby reducing their confidence in using their backs.^31^ An observational study linked kinesiophobia and catastrophic thoughts about pain to more extended work absences and disability among back pain patients in occupational insurance contexts.^42^ Our findings can be interpreted through the activity and fear-avoidance models, which show that pain can lead to avoidance and catastrophizing behaviors.^41^ High fear-related beliefs from painful experiences can result in escape and avoidance actions, increasing physical and social inactivity.^41^ This fosters mood disturbances such as irritability and frustration, disuse, and chronic pain, worsening treatment outcomes.^41^ Negative attitudes towards physical activity elevate disability rates, with avoidance strategies adversely affecting recovery.^43^ Back-PAQ and FABQ-Physical Activity assess partly overlapping beliefs (r=0.339, n=269); their association may partly reflect shared content and common-method variance.

A longer duration of LBP was independently associated with more negative attitudes toward LBP. People who experience symptoms for longer periods can form more negative thoughts about symptoms going away, feel that symptoms are permanent and feel more worried about having more symptoms in the future. The pain that occurs repeatedly, along with failed treatment and persistent functional impairments, could perpetuate a fear of back vulnerability and limited healing. This result aligns with previous evidence that suggests that unhelpful beliefs may become more entrenched over time and play a role in the chronicity of LBP.^10^,^16^ Longitudinal research is needed before inferring that beliefs become more unhelpful as symptoms persist. Duration was analyzed continuously to avoid arbitrary grouping, but its association was not significant after log transformation.

Understanding negative attitudes may inform future interventions to manage LBP.^44^ Cognitive therapy, mindfulness, and motivational interviewing are possible subjects for future intervention research. Intervention trials are needed to determine whether educating patients about LBP and addressing their beliefs improves rehabilitation or recovery.

## Strengths and Limitations

This was a cross-sectional study; thus, the significant associations observed between independent and dependent variables indicate only correlations, not causation. A strength of this study is that it included an adequate sample of adults receiving physiotherapy for LBP from multiple healthcare sectors and geographic regions in Jordan. The study also used standardized Arabic measures of back-pain attitudes, fear-avoidance beliefs, psychological distress, pain intensity, and disability. However, several limitations should be considered. First, a cross-sectional design prevents conclusions about directionality or causality. Second, participants were recruited from physiotherapy settings; therefore, the findings may not generalize to the wider community, to people with LBP who do not seek care, or to patients managed exclusively in primary care. Third, all questionnaire data were self-reported and may be affected by recall bias or social desirability. Fourth, although we measured the FABQ-Work subscale, its relevance may vary among unemployed participants or those whose symptoms were not work-related. Finally, the study examined factors associated with Back-PAQ scores. It did not test whether Back-PAQ, FABQ, DASS, or ODI scores predict future pain, disability, or clinically meaningful change over time. The response rate is not reported, and non-participation bias cannot be estimated because the samples are non-probability samples. Social-desirability effects may be greater when interviewers conduct interviews than when participants self-administer them, especially if interviewers appear to be care providers. Selection bias may result from “complete case” analysis. The complete sample is identical to the one previously published in the knowledge study,^19^ and the present report is not an independent replication of that sample. The 2019–2021 recruitment period overlapped the COVID-19 pandemic, when access to care and clinic patient demographics may have shifted. Unmeasured clustering within clinics may also affect precision because a verified recruitment-site identifier was unavailable, and interviewers’ care-provider roles were not documented. These documentation gaps limit reproducibility.

Further longitudinal studies are warranted to determine whether negative beliefs measured by Back-PAQ predict future pain intensity, disability, care-seeking, or response to physiotherapy. Further intervention trials are also needed to determine whether interventions addressing activity-related fear avoidance and back vulnerability beliefs can improve attitudes, disability and long-term self-management outcomes.

## Conclusion

Adults receiving physiotherapy for LBP in this non-probability clinical sample endorsed beliefs about their condition, particularly beliefs that the back is vulnerable, that pain indicates harm, and that activity should be restricted during pain episodes. In the multivariable model, higher Back-PAQ total scores were associated with female gender, longer LBP duration, moderate pain intensity, and higher fear-avoidance beliefs related to physical activity. The duration association and moderate-pain contrast were sensitive to analytic choices. These cross-sectional associations do not establish directionality, screening accuracy, prognosis, or the effectiveness of education or other interventions.

## Data Availability

The data that support the findings of this study are available from the corresponding author upon reasonable request. The data are not publicly available due to ethical and privacy restrictions related to participant confidentiality.

## Generative Artificial Intelligence (AI) Statement

The authors used ChatGPT (OpenAI, GPT-5.5 Thinking) to assist with language editing, grammar, clarity, and readability during manuscript preparation. The authors reviewed and verified all AI-assisted content and take full responsibility for the final manuscript. No AI tool was used to generate data, perform analyses, or create references.

## Abbreviations

Back-PAQ: Back-Pain Attitudes Questionnaire; DASS: Depression, Anxiety, and Stress Scales; FABQ: Fear-Avoidance Beliefs Questionnaire; LBP: low back pain; NPRS: Numeric Pain Rating Scale; ODI: Oswestry Disability Index.
